# Development of Clinical Vignettes to Describe Alzheimer's Disease Health States: A Qualitative Study

**DOI:** 10.1371/journal.pone.0162422

**Published:** 2016-09-02

**Authors:** Mark Oremus, Feng Xie, Kathryn Gaebel

**Affiliations:** 1 School of Public Health and Health Systems, University of Waterloo, Waterloo, Ontario, Canada; 2 Department of Clinical Epidemiology and Biostatistics, McMaster University, Hamilton, Ontario, Canada; 3 Program for Health Economics and Outcome Measures (PHENOM), Hamilton, Ontario, Canada; 4 Centre for Evaluation of Medicines of St. Joseph’s Healthcare Hamilton, Hamilton, Ontario, Canada; Xuanwu Hospital, Capital Medical Universty, CHINA

## Abstract

**Aims:**

To develop clinical descriptions (vignettes) of life with Alzheimer’s disease (AD), we conducted focus groups of persons with AD (n = 14), family caregivers of persons with AD (n = 20), and clinicians who see persons with AD in their practices (n = 5).

**Methods:**

Group participants read existing descriptions of AD and commented on the realism and comprehensibility of the descriptions. We used thematic framework analysis to code the comments into themes and develop three new vignettes to describe mild, moderate, and severe AD.

**Results:**

Themes included the types of symptoms to mention in the new vignettes, plus the manner in which the vignettes should be written. Since the vignette descriptions were based on focus group participants’ first-hand knowledge of AD, the descriptions can be said to demonstrate content validity.

**Conclusion:**

Members of the general public can read the vignettes and estimate their health-related quality-of-life (HRQoL) as if they had AD based on the vignette descriptions. This is especially important for economic evaluations of new AD medications, which require HRQoL to be assessed in a manner that persons with AD often find difficult to undertake. The vignettes will allow the general public to serve as a proxy and provide HRQoL estimates in place of persons with AD.

## Introduction

Alzheimer’s disease (AD) is a neurodegenerative disorder that is characterized by progressive declines in cognitive and functional abilities. Early symptoms include a loss of short-term memory, immediate event recall, and attention. Persons with AD (PwAD) may also experience disorientation or depression. Over time, these persons lose the ability to perform instrumental activities of daily living, including preparing meals, managing money, shopping, performing housework, and using a telephone. In the later stages of the disease, PwAD lose the ability to perform basic activities of daily living, which include bathing or showering, dressing, getting in and out of bed or a chair, using the toilet, and eating [1].

The clinical presentation of AD varies widely. Health-related quality-of-life (HRQoL) is a useful common metric for measuring the differential impact of AD across individuals [2]. Valid HRQoL estimates are needed to conduct economic evaluations of new AD treatments. These evaluations are becoming ever more important components of public health insurance reimbursement decisions in AD [3,4]. However, evidence suggests many PwAD experience difficulties estimating their own HRQoL [5,6] on instruments such as the EQ-5D-5L [7], which provide the type of data needed for economic evaluations. When family caregivers are used as proxies to estimate the HRQoL of PwAD, they often provide underestimates because they integrate their own life experiences (e.g., the burden and stress of caregiving) into the proxy assessments [8–14]. We are embarking upon a research program to examine whether the general public can provide valid proxy health-related quality-of-life (HRQoL) estimates in place of PwAD and their caregivers. To date, the potential role of the general public in this respect remains largely unexplored.

Since most members of the general public are unlikely to have first-hand knowledge of AD, we will use clinical vignettes to present the general public with descriptions of the mild, moderate, and severe health states of the disease. These persons will read the vignettes and answer the EQ-5D-5L as if they had AD according to the vignette descriptions.

### Clinical Vignettes

According to Alexander and Becker [15], clinical vignettes are “short descriptions of a person or a social situation which contain precise references to what are thought to be the most important factors in the decision-making or judgment-making process of respondents”. Vignettes are used extensively in AD research to study diverse issues such as treatment preferences, emotional reactions to the disease, and HRQoL [16]. AD researchers usually employ a type of vignette design called ‘paper people studies’ in their work. This design requires study participants to read descriptions of people or situations and make decisions, judgments, or choices based on the descriptions [17]. For example, a recent study presented 789 American adults with a series of vignettes describing the signs and symptoms of mild AD [18]. The researchers attached different disease labels to the vignettes and examined whether participants developed stigmatizing reactions to AD based on the labels or the signs and symptoms of the disease.

Despite the frequent use of vignettes in AD research, only a minority of published articles describe how researchers developed the vignettes; some articles do not even reproduce the text of the vignettes [16]. This lack of detail creates difficulty for readers who wish to assess whether the vignettes used in the research possess content validity.

Since the general public’s proxy HRQoL estimates in AD will flow from reading clinical vignettes, we conducted a qualitative study to develop a set of vignettes with content validity. Content validity is the extent to which the vignettes represent the most salient features of a concept such as AD [19]. Qualitative content validation of the vignettes can be accomplished using different groups of informants to provide a rich set of perspectives on AD [20]. These groups can include PwAD and their caregivers to provide an insider’s (emic) perspective on the content of the vignettes and clinicians to provide an outsider’s (etic) perspective [20]. This manuscript details the qualitative development of the vignettes. The vignettes may be used by other researchers who require study participants to read descriptions of AD.

## Materials and Methods

### Participants

To develop the vignettes, we organized a series of focus groups composed of clinicians who see PwAD in their practices, PwAD, and the family caregivers of PwAD. We ran separate focus groups for each type of individual because we felt that mixed groups would discourage the free flow of ideas. For example, PwAD or their caregivers might be reluctant to ‘speak up’ in front of a doctor, or caregivers might not describe their full experiences with AD to avoid upsetting a loved one who is attending the same focus group.

The clinicians were recruited because of their experience treating PwAD and conducting AD research. The clinicians had previously collaborated with the lead author [21], or they were referred to the lead author by other clinicians. We used the patient lists from three memory or geriatric clinics in Toronto (Canada) to identify persons with mild or moderate AD and their family caregivers. The physicians in the clinics diagnosed disease and assessed severity using the standards in place at their respective locations, e.g., Diagnostic and Statistical Manual of Mental Disorders, 5th Edition [22], Institute of Aging and Alzheimer’s Association criteria [23], Functional Assessment Staging Test [24]. Clinic staff telephoned the caregivers, explained the study, and attempted to recruit both the caregivers and their family members with AD. To participate, individuals had to understand, speak, and read English. We included persons with mild or moderate AD to maximize the potential of recruiting individuals who could participate in interactive group settings.

### Focus Groups

At the start of each focus group, participants read a vignette describing mild AD [25]. The focus group facilitator used a semi-structured interview guide (S1 File) to ask the participants whether we should add or remove descriptions of disease symptoms from the vignette, whether the examples used in the vignette to explain the symptoms actually captured the average person’s experience with AD, and whether the wording used in the vignette was clear and concise. Once the group exhausted all discussion of the points raised in the interview guide, the facilitator repeated the process for vignettes that described moderate and severe AD [25].

The vignettes used to start the focus group discussions came from our earlier research [25]. We developed these earlier vignettes using published information [26–28] and descriptions of AD [29–31]. However, we did not establish the content validity of the earlier vignettes.

### Analysis

The focus groups were audio recorded and transcribed verbatim. We used thematic framework analysis [32] and NVIVO v10 (QSR International Pty Ltd, Doncaster, Australia) to derive themes and sub-themes from the written transcripts. Two coders independently read each transcript. While reading the first transcript, the coders segmented and labeled the text into a set of preliminary codes. The codes were revised iteratively as each coder read additional transcripts. During the iterative process, similar codes were aggregated together into themes. As the process continued, the coders created new themes, combined similar themes, and placed groups of related themes (sub-themes) together under larger themes. Once the coding was complete, the coders reviewed each other’s work and arrived at a common set of themes and sub-themes through consensus.

The common set of themes and sub-themes was a summary of the focus groups’ deliberations [25]. We used this summary to substantively revise the original vignettes that had been presented to the focus groups for discussion. The revisions were substantive enough to produce what was essentially a new set of vignettes. The ‘new’ vignettes are available for viewing in an open access format [33].

### Ethics

The study received ethics approval from the Hamilton Integrated Research Ethics Board (study #: 13–271), Research Ethics Board of Sunnybrooke Health Sciences Centre (study #: 230–2013), Baycrest Research Ethics Board (study #: 13–48), and St. Michael’s Hospital Research Ethics Board (study #: 13–198). Participants gave written informed consent prior to the start of the focus groups. During the informed consent process, participants were told orally and in writing that anonymized quotes from the focus group discussions could be used in published articles. Physicians from the recruitment clinics recruited persons with mild or moderate AD who maintained the cognitive capacity to consent to research and participate meaningfully in focus group discussions. Proxy consent was not required for any PwAD.

### Article Reporting

We used the consolidated criteria for reporting qualitative research (COREQ-32) [34] to summarize important aspects of the study in tabular form (Table 1).

**Table 1:** COREQ-32 checklist.

Domain 1: Research team and reflexivity	
*Personal characteristics*	
Interviewer/facilitator	Mark Oremus (MO) / Feng Xie (FX) / Kathryn Gaebel (KG)
Credentials	PhD / PhD / MSc
Occupation	Professor / Professor / Research Associate
Gender	Male / Male / Female
Experience and training	Academic researcher / Academic researcher / Professional interviewer and research coordinator in academic hospital
*Relationship with participants*	
Relationship established	MO conducted previous research with four of the clinicians in the clinician focus group; FX and KG did not know any of the clinicians; MO, FX, and KG had no prior relationships with any of the persons with Alzheimer’s disease or caregivers
Participant knowledge of the interviewer	Four clinicians collaborated with one of the interviewers (MO) on a previous Alzheimer’s disease research project; none of the persons with Alzheimer’s disease or caregivers knew any of the interviewers prior to the focus groups
Interviewer characteristics	No characteristics were reported
Domain 2: Study design	
*Theoretical framework*	
Methodological orientation and theory	Thematic framework analysis
*Participant selection*	
Sampling	Clinicians: purposive, snowball; persons with Alzheimer’s disease and caregivers: purposive, convenience
Method of approach	Clinicians: e-mail; persons with Alzheimer’s disease and caregivers: face-to-face
Sample size	7 clinicians; 14 persons with Alzheimer’s disease; 20 caregivers
Non-participation	No participants dropped out. All clinicians who were asked to participate agreed to do so; 21 persons with Alzheimer’s disease and 15 caregivers did not participate for various reasons (too ill, too busy, not interested, scheduling conflicts)
*Setting*	
Setting of data collection	Meeting room in a university (clinician focus group); meeting rooms in hospitals (persons with Alzheimer’s disease and caregiver focus groups); telephone (two one-on-one interviews with clinicians)
Presence of non-participants	No
Description of sample	See Table 2
*Data collection*	
Interview guide	The authors wrote the questions and prompts were given during the interviews if needed. No pilot testing.
Repeat interviews	No
Audio/visual recording	Audio recording and verbatim transcription
Field notes	No
Duration	Focus groups: average duration 90 minutes (including administration of informed consent); telephone interviews: 30 minutes
Data saturation	MO and KG debriefed each other following each focus group and decided when data saturation occurred.
Transcripts returned	No
Domain 3: Analysis and findings	
*Data analysis*	
Number of data coders	Two (MO, KG)
Descriptions of the coding tree	Yes–three themes and 20 sub-themes (see Table 3)
Derivation of themes	Data-derived themes
Software	nVIVO v10
Participant checking	No
*Reporting*	
Quotations presented	Yes–quotations were identified by category (i.e., clinician, person with Alzheimer’s disease, caregiver)
Data and findings consistent	Yes
Clarity of major themes	Yes–three major themes are reported in this publication
Clarity of minor themes	Yes– 20 sub-themes are reported in this publication

## Results

### Focus Groups and Sample Characteristics

We completed seven focus groups. Three focus groups included persons with mild or moderate AD (14 persons total), three separate groups included the family caregivers of persons with mild or moderate AD (20 caregivers total), and one separate group included clinicians (two geriatricians, two neuropsychiatrists, and a clinical researcher). Fourteen of the caregivers were the spouses or children of the 14 PwAD who were included in the study. We interviewed two other clinicians (geriatrician, geriatric psychiatrist) via telephone because they were unable to join the clinicians’ focus group. We used the semi-structured interview guide from the focus groups to conduct the telephone interviews.

Input from the final two focus groups (one of PwAD, one of caregivers) was markedly similar to the input from the previous five focus groups, regardless of participant type, thereby suggesting we had achieved saturation and could cease further recruitment [35].

The median age of all 41 qualitative study participants was 70 years and 20 were female (Table 2). Among the PwAD and the caregivers, almost half (n = 16) were university graduates and most (n = 19) reported annual household incomes of at least $80,000. All of the clinicians were university graduates with incomes of at least $80,000.

**Table 2:** Sample characteristics.

Characteristic	Data
*Age–median (25*^*th*^*– 75*^*th*^ *percentiles)*^a^	70.0 y (59.0 y– 79.0 y)
*Sex–n*^a^	
Female	20
Male	21
*Education–n*^b^	
Less than high school	2
High school graduate	5
Technical or trade school graduate	4
College graduate	7
University graduate (Bachelor, Master’s, Doctorate)	16
*Annual household income*^c^ *–n*^b^	
< $20,000	2
$20,000 - $39,999	1
$40,000 - $59,999	4
$60,000 - $79,999	4
≥ $80,000	19
Missing	4

n, number of participants; y, years.

^a^ Data includes all 41 qualitative study participants.

^b^ Data includes 14 persons with Alzheimer’s disease and 20 caregivers of persons with Alzheimer’s disease (all clinicians were university graduates with incomes ≥ $80,000).

^c^ Canadian dollars.

### Themes and Sub-themes

We extracted three themes (and 20 sub-themes) from the transcripts of the focus groups and one-on-one interviews. The themes (sub-themes in brackets) included symptoms (apathy, aggression, concentration, confusion, daily living, decision making, memory, mobility, personality, repeating, shadowing, social interaction, wandering, and word finding), adaptation to living with AD (use of notes and prompts, language, inability to adapt), and format of vignettes (use of examples, wording, paragraph versus point form). Table 3 contains representative participant quotes for each sub-theme.

**Table 3:** Focus group themes and sub-themes.

Theme		Focus group participant quotes
	Sub-theme	
Adaptation		
	Use of notes and prompts	*“If I go to a store I will write it down*.*” [Mi] [PwAD]*
		*“I walked into her [mother’s] condo and she had notes strewn all over the floor*, *like I couldn’t’ make heads or tails out of anything*. *You know*, *she wrote the same note maybe 10 times*. *And so I would walk around and I said why are you doing this and she said oh I have to remember*.*” [Mi] [CG]*
		*“I also have a calendar*, *it’s like a book*, *so if I have to go someplace I write it in the*, *I put it in there and when it’s finished and I am past November the 1st I cross it off*. *I am not at the end of November I think*, *so I try and solve everything as I go along*, *and I always know the date too*. *You have tricks*.*” [Mo] [PwAD]*
	Language	*“My wife is very good at disguising her disease to people*, *she is using very general sentences*, *as long as we are happy*, *it fits every discussions*.*” [Mi] [CG]*
	Inability to adapt	*“But by the time they need the yellow sticky notes all over the place*, *they can’t remember to look at the yellow sticky note or where they found it*.*” [Mi] [CL]*
		*“I just saw a person yesterday in clinic who was clearly just taking reams of notes about stuff*. *But one of the keys to that was despite taking those notes*, *I think this comes to what you mentioned earlier she would never really be able to remember to refer to them*. *The notes that she would take would not always make sense*, *they were very*, *very detailed about everything*, *she was trying to write down everything so that she could remember*. *But it wasn’t really accomplishing at all what she wanted to do*.*” [Mo] [CL]*
Format of vignettes		
	Use of examples	*“So could we say you might not remember an event of importance for you*, *for example you regularly*, *you might forget Team Canada when you watch the games*. *Or just say something important to you to que the person who is reading it that this is supposed to be something important to you*. *So take that one example and just say you might not remember something that was important to you for example*.*” [All vignettes] [CL]*
		*“You know*, *I think the examples are certainly reasonable*. *Of course it comes to the fact there are some people who will*, *certain people just don’t follow for instance hockey*, *or you know or Olympic sports and stuff like that*, *some people don’t necessarily follow politics*. *I am not sure there’s going to be one*, *any one example that’s going to be you know*, *inclusive of all people’s experiences*. *So*, *I mean I thought when I was reading through those were just fine with regards to things*. *The issue I suppose is the timing when you introduce this too as well and of course Team Canada*, *Olympic Gold Hockey Medal those are certainly married with the Winter Olympics for example*. *And it’s been awhile since the Winter Olympics*, *I guess it just really depends when that’s introduced*. *Also as well it depends on how long you are going to I guess span*, *this*, *this study only in the context of if for instance any of the information changes you know*, *the Winter Olympics coming up again in 2014 right*, *in Russia*. *So it just*, *other than that however*, *I wouldn’t really have a problem with the examples that you have given*.*” [All vignettes] [CL]*
	Wording	*“I like the fact that you put qualifiers [words like ‘may’*, *‘might’] in for most of this and then there are other areas where you don’t [use of definitive words like ‘will’] and I wonder why*, *why that shifted*. *So you might forget is helpful and some may not but then you will still be aware of the day and time*.*” [All vignettes] [CL]*
	Paragraph versus point form	*“I like the paragraph as opposed to the point form*, *more narrative to me of what the story is*.*” [All vignettes] [CL]*
Symptoms		
	Apathy	*“My dad he has stopped going to the Seniors club and he didn’t want to talk on the phone*, *and he started withdrawing himself*.*” [Mo] [CG]*
		*“Loss of enjoyment of their social activities and what they do*. *I mean often you hear we took dad out to dinner and after being there for 10 minutes you just want to go home*.*” [Mo] [CL]*
	Aggression	*“I saw the extreme in both my parents in different ways*, *biting*, *kicking*, *my father would yell at people in the restaurant if they didn’t bring the right thing*.*” [S] [CG]*
	Concentration	*“I find this too*, *my husband watches the news*, *watching*, *watching but then after if I try commenting on something he says nothing*. *So I don’t think he’s really concentrating and really following what it’s all about*. *Spent his whole life reading but now doesn’t read at all because I think he can’t concentrate*.*” [Mi] [CG]*
		*“On concentration my husband loves to read the newspaper for an hour or two for a good part of his life*. *But I will come home from shopping and he’s been sitting at the table and he’s only gone through about three pages*.*” [Mi] [CG]*
	Confusion	*“She doesn’t think to go into*, *to look at a cupboard even below her sink to get out another toilet paper roll*. *She will call me and say I’m out of toilet paper and there is 10 of them underneath or Kleenex*, *that sort of thing*.*” [Mo] [CG]*
		*“Regardless of whether or not you are in let’s say a complex care facility or versus brought to the family members home for a special dinner or something like that or going to a local shopping mall*. *There may be just that general lack of awareness of where you are and you know*, *what the appropriate sort of behaviors would be in that circumstance*.*” [S] [CL]*
	Daily living	*“I guess the question is do you want to put in some positive things*, *you may still enjoy a good meal and treats*. *Ah*, *you still enjoy*, *you might still enjoy singing*.*” [S] [CL]*
		*“You will need full-time help with practically everything that you would do during the course of your day*, *such as eating*, *dressing*, *bathing*, *toileting*.*” [S] [CL]*
		*“No*, *well I mean*, *really it’s just basically people at that level and this is how I visualize it*, *their basic activities*. *Things that you highlighted would be eating*, *dressing*, *grooming*, *bathing*, *toileting*, *mobility*, *often they have difficulties with regards to balance*. *No*, *I think those are the main things that I would you know*, *really want to highlight*.*” [S] [CL]*
		*“If you live at home you are going to need full-time help*, *if not then you are going to have to be living somewhere else*.*” [S] [CL]*
	Decision making	*“With my mom one of the things we notice was difficult with making decisions*, *even simple things like being in a restaurant*, *talking her out for a meal and we would be looking at the menu*. *And I would say mom what is it you would like*, *and she would say well what are you having*? *And it was always I’ll have what you’re having*, *you know*, *something as simple as that*.*” [Mi] [CG]*
	Memory	*“I think this ah*, *in my case*, *she doesn’t know what day of the week it is most of the time*. *But she does remember the names of her children and grandchildren pretty well*. *Some of the people that might drop into the house she wouldn’t know who they were*, *she would say why did they come*, *whatever*, *and things like that*. *And she ah*, *well I think she knows whether it’s summer or winter basically*, *she knows*, *maybe not the date or what month it is maybe*.*” [Mo] [CG]*
		*“You’ve forgotten significant life events*, *like who you were married to and things like that*.*” [S] [CG]*
		*“You are somewhere but you don’t know where you are*, *you don’t know where and time or what the time is or what the season of the year is*, *you don’t know anything like that*. *You just know kind of who you are and what your name is*.*” [S] [CG]*
	Mobility	*“But one of the things I did notice and nobody has mentioned it*, *my mom was always extremely active and then I started seeing that she had stability and balance issues*.*” [Mi] [CG]*
	Personality	*“My husband is the opposite*, *he never used to like to dance*, *like he would dance now we go out dancing he’s the life of the party*. *He will fool around with waitresses*, *he will joke with them*, *he’s become very social more so then ever*.*” [Mo] [CG]*
	Repeat	*“Hello*, *how are you*, *and I would say fine dad*, *how are you and he would say fine*. *Then five minutes after that how are you*, *I would go fine dad how are you*, *fine*. *And then he would be like sitting there*, *five minutes after*, *how are you*, *okay*, *here we go again*, *fine*.*” [Mi] [CG]*
		*“On that subject*, *sitting in the doctor’s and a couple came in with a mother and it seemed quite clear to me that she had Alzheimer’s and she kept asking the same question*, *a couple of questions over and over again*. *They were very good with her and all of a sudden the man*, *I guess he got fed up and he said will you stop asking me questions and I mean it*.*” [Mo] [CG]*
		*“Only few people that are really perceptive can see that she is repeating herself*.*” [Mi] [CG]*
	Shadowing	*“That’s another thing*, *they rely a lot*, *like when we are out*, *he will always say to me go ahead*, *when we are leaving a store he will say go ahead so he doesn’t have to find the car he will follow me*.*” [Mi] [CG]*
	Social interaction	*“Right*, *so things like that*. *Um*, *no filter when she is speaking*. *And also I don’t know if anybody else experienced this but my mom*, *my mom would start talking so inappropriate*, *like the conversation was all sexual and I would like crawl under the table and want to die*. *We would go to a restaurant and I would look at her and say you can’t*, *you can’t say that*, *she just didn’t care*, *she didn’t care*.*” [Mi] [CG]*
		*“And spitting*, *you know*, *from a person who had such incredible manners from then to not looking for a Kleenex*, *you know you might have a little bit of phlegm or something and spitting into flower boxes*. *Just those kinds of things*, *losing that decorum you know was interesting as an addition for the moderate*.*” [Mo] [CG]*
	Wandering	*“If you take her to a shopping mall you’ve got to watch her because she will get lost*.*” [Mi] [CG]*
		*“Wandering around at night is common*, *pacing*.*” [S] [CG]*
	Word finding	*“One of the things you should probably mention is word finding difficulties because many patients will complain about that*.*” [Mi] [CL*

All vignettes, quote applies to all three vignettes; CG, quote comes from a caregiver; CL, quote comes from a clinician; Mi, quote applies to mild Alzheimer’s disease vignette; Mo, quote applies to moderate Alzheimer’s disease vignette; PwAD, quote comes from a person with Alzheimer’s disease; S, quote applies to severe Alzheimer’s disease vignette.

The sub-themes applied to specific vignettes or to all three vignettes. An example of a sub-theme that applied to a specific vignette was ‘use of notes and prompts’. Many participants discussed the use of notes or calendars to adapt to the memory challenges posed by AD. In the vignette for mild AD, we captured this feedback by adding the following sentence: “You may need to post notes around the home to remind you of simple things like turning off the stove.” Another sub-theme, ‘inability to adapt’, suggested that persons with moderate or severe AD would no longer be able to implement effective adaptation strategies. Therefore, we did not provide examples of adaptation in the vignettes for moderate or severe AD.

Turning to an example of a sub-theme that applied to all three vignettes, the ‘wording’ sub-theme suggested qualifier words such as ‘may’ or ‘might’ (rather than definitive words such as ‘will’) should be used to describe life with AD. We ensured the qualifier words were employed throughout the text in all three vignettes. For example, in the severe vignette, we wrote “You *would* probably need full-time help with what [tasks] you cannot do…”, instead of “You *will* need full-time help…”. Qualifiers account for the fact that the clinical presentation of AD varies widely. No one mix of symptoms applies to all persons with the disease. For members of the general public who know PwAD, the qualifiers maintain the generality of the vignettes. Individuals will not dismiss the vignettes if the text fails to mirror real-life experiences with AD.

The ‘daily living’ sub-theme also applied to all three vignettes. Focus group participants suggested we show that life with AD is not entirely dour. We added text to say that PwAD might enjoy puzzles or reading (mild vignette), listening to music or watching television (moderate vignette), or eating certain foods (severe vignette). Table 4 provides a broader list of examples to illustrate how focus group feedback built the content of the vignettes.

**Table 4:** Examples of content contributions to vignettes based on focus group themes and sub-themes.

Theme		Content contribution
	Sub-theme	
Adaptation		
	Use of notes and prompts	Added the following description to the mild vignette: “You may need to post notes around the home to remind you of simple things like turning off the stove.”
	Language	No contribution to the vignettes because language primarily affects people who interact with persons with AD. However, the persons themselves are unlikely to perceive any deleterious effects on HRQoL as a result of language or repetition issues.
	Inability to adapt	No means of adaptation to living with AD (e.g., notes or prompts) included in the moderate and severe vignettes.
Format of vignettes		
	Use of examples	No example applies to everyone, but generic examples can help readers of the vignettes understand the practical implications of having AD. Generic examples added to the vignettes included “You may not follow the flow of conversations in social gatherings” [Mo] and “You would probably need full-time help with what you cannot do and you would be unable to live alone” [S].
		Some examples were written to be generic. In the vignettes from our earlier work [25], one example stated that persons with AD would not remember who won the Olympic gold medal in hockey, even though they watched the game. We re-wrote the example to read “…if you are interested in hockey, then you might forget whether your favorite team won their last game, although you may remember watching the game itself” [Mi].
	Wording	Since the clinical presentation of AD varies from person to person, the vignettes included qualifiers such as ‘may’ or ‘might’ throughout the text, e.g., “…you may be unable to do one or more of several basic chores on your own, including eating, dressing, bathing, toileting, looking after personal hygiene, or walking” [S].
	Paragraph versus point form	The vignettes were written in paragraph form.
Symptoms		
	Apathy	Added the following description to the moderate vignette: “You may lose interest in doing many of the things that you once liked to do.”
	Aggression	Added the following description to the severe vignette: “You may experience agitated behaviour, such as struggling with the person who is trying to bathe or dress you, even if this person is your spouse or child.”
	Concentration	Added the following description to the mild vignette: “Your ability to concentrate may decrease….”
	Confusion	Added the following description to the moderate vignette: “You might misplace common items like your glasses or the toothpaste.”
		Added the following description to the severe vignette: “…you may be unaware of where you are.”
	Daily living	Added the following descriptions to each of the vignettes, e.g., “You may no longer be able to handle your personal finances or plan leisure activities such as dinner parties or vacations” [Mi], “You would need help with things like choosing what clothing to wear, using the telephone or computer, paying bills, cleaning house, buying groceries, preparing meals, or taking medications” [Mo], “You may be unable to do one or more of several basic chores on your own, including eating, dressing, bathing, toileting, looking after personal hygiene, or walking. You would probably need full-time help with what you cannot do and you would be unable to live alone” [S].
		Positive aspects of daily living were also added to the vignettes, e.g., “You may continue to enjoy hobbies such as puzzles or reading” [Mi], “You may still enjoy activities like listening to music or watching television” [Mo], “You may enjoy certain foods or passive activities like listening to music” [S].
	Decision making	Added the following description to the mild vignette: “Simple decisions such as what to order in restaurants might become difficult to make.”
	Memory	Added the following description to the moderate vignette: “…you might forget or mix up the names of your grandchildren or in-laws, but you may remember the names of your spouse and children. Sometimes you may not know the date or day of the week.”
		Added the following description to the severe vignette: “You may not know the day, date, or season of the year, and you may be unaware of where you are.”
	Mobility	Added the following description to the mild vignette: “You might be able to travel to familiar locations like the supermarket, although probably with the help of a loved one or friend.”
	Personality	Added the following description to the moderate vignette: “You may experience behavior and mood changes, such as becoming outgoing and talkative if you were once shy, or vice versa.”
	Repeat	No changes made to the vignettes because repeating primarily affects people who interact with persons with AD. However, the persons themselves are unlikely to perceive any deleterious effects on HRQoL as a result of repeating words or sentences.
	Shadowing	No changes made to the vignettes because shadowing primarily affects people who interact with persons with AD. However, the persons themselves are unlikely to perceive any deleterious effects on HRQoL as a result of shadowing.
	Social interaction	No changes made to the vignettes because social interaction primarily affects people who interact with persons with AD. However, the persons themselves are unlikely to perceive any deleterious effects on HRQoL as a result of how they interact with others.
	Wandering	Added the following description to the moderate vignette: “If you do go out, then someone might have to guide you the entire time.”
	Word finding	Added the following description to the mild vignette: “You may start having trouble finding words to express your thoughts.”

AD, Alzheimer’s disease; HRQoL, health-related quality-of-life; Mi, applies to mild Alzheimer’s disease vignette; Mo, applies to moderate Alzheimer’s disease vignette; S, applies to severe Alzheimer’s disease vignette.

We did not utilize four sub-themes to inform vignette content, i.e., language (e.g., speaking in general sentences), repeating sentences or questions, shadowing (i.e., following caregivers in rote fashion without purpose), and social interaction (e.g., making inappropriate comments or performing inappropriate actions in public). These sub-themes describe behaviors that would more likely affect people who interact with PwAD, rather than the PwAD themselves. Therefore, these behaviors would be unlikely to affect the HRQoL of PwAD.

## Discussion

The vignettes that emerged from the focus group discussions contained practical examples—based on group members’ first-hand knowledge of AD—to describe the broad spectrum of symptoms that PwAD might experience daily. The purpose of structuring the vignettes in this fashion was to create straightforward lay descriptions of AD and enable the general public (most of whom would be unfamiliar with the specifics of the disease) to gain an awareness of what life is like with the condition. We felt such an awareness would enhance the general public’s ability to provide proxy HRQoL estimates in place of PwAD.

A recent review of vignette-based studies in AD [16] found that many existing vignettes were crafted to meet specific study objectives, e.g., driving and dementia [36]. Unlike the vignettes in this study, the existing vignettes do not broadly describe what life is like with AD. The vignettes from this study can be used in research that requires participants to have a comprehensive perspective on the manner in which AD affects people’s lives. The comprehensive perspective is useful not only for economic evaluations, but also for health services research undertaken to elicit the general public’s revealed preferences for resource allocation (e.g., should the healthcare system assign greater resources to AD versus cardiovascular disease?). From a policy perspective, a means for the general public to provide input into healthcare resource allocation decisions is necessary because the public utilizes health services and finances such services in many healthcare jurisdictions (e.g., Canada, Western Europe) [37].

### Strengths and Limitations

Compared to one-on-one interviews, focus groups provide a forum for participants to build upon one another’s comments and generate richer input. Additionally, focus groups can identify unusual or outlier opinions that might otherwise pass unnoticed in one-on-one interviews [38,39]. These focus group characteristics were especially important in our study because we wanted the vignettes to describe a broad range of common AD symptoms to account for the heterogeneous clinical presentation of the disease. The group environment helped us establish a consensus about common symptoms and permitted us to exclude less common symptoms.

The focus group discussions served as a means of promoting vignette equivalence [40]. Vignette equivalence is the degree to which people interpret vignette descriptions similarly to one another. Researchers may access equivalence by changing components of the text (e.g., “…*will* experience forgetfulness…” to “…*may* experience forgetfulness…”) and examining whether people’s interpretations of the vignettes would subsequently change. When the issue of using qualifier instead of definitive words arose in the first focus group, we specifically asked participants in the following groups whether this wording would affect their interpretation of the vignettes. The consensus, both within and across groups, was that qualifier words would maximize the public’s acceptability of the vignettes, as we discussed above. Conversely, the focus groups felt definitive words would foster rigid (‘take it or leave it’) descriptions of AD that might not resonate with people who know someone with the disease.

The vignettes demonstrated response consistency [41] in a quantitative study [33] that followed this qualitative study. A sample of the general public rated their own current HRQoL and read the vignettes to estimate what their HRQoL would be like with AD. Participants’ average HRQoL estimates for mild AD were statistically significantly lower than the average HRQoL ratings for their current health states. Furthermore, average HRQoL estimates were statistically significantly lower for moderate versus mild AD and severe versus moderate AD [33].

We did not employ member checking—asking members of the original focus groups to review the vignettes and ensure we interpreted their input correctly—to verify the accuracy of our qualitative work. Recent methods guidance advises against member checking because researchers may not be able to determine whether participants who provide negative feedback during member checking have identified interpretation issues or simply changed their minds over time [42].

Most of the quotes that provided enough detail to inform the content of the vignettes came from caregivers and clinicians. While we recruited persons with mild or moderate AD to obtain the perspective of individuals who are most directly affected by the disease, the quotes from PwAD were frequently anecdotal and too sparse to inform vignette content on their own. For example, one PwAD lengthily described a difficult turn he made during a road test taken to maintain driving privileges. Another PwAD reported picking up orange juice instead of bananas at the grocery store. These anecdotes were useful to confirm the relevance of sub-themes such as ‘daily living’ and ‘memory’, which emerged out of caregivers’ and clinicians’ much more structured and focused input. Since the vignettes’ content was guided by caregivers and clinicians, the vignettes may not have captured components of life with AD that could only be perceived by persons with the disease, but which these persons could not articulate due to the effects of cognitive impairment.

We did not include paid caregivers in the study. These individuals typically work for homecare providers or long-term care facilities. Paid caregivers usually enter the circle of care for PwAD after the disease has progressed to more severe health states. Clinicians were more likely to posses experience with PwAD over the entire spectrum of disease severity. However, paid caregivers and other health professionals such as nurses might possess insights into life with AD that differ from the clinician’s perspective. The vignettes did not capture these insights.

### Future Research

An important means of assessing vignette validity would be to ask PwAD to estimate their own current HRQoL on the EQ-5D-5L and subsequently read the vignettes and answer the EQ-5D-5L as if they had AD based on the vignette descriptions. Responses to both administrations of the EQ-5D-5L could then be compared to examine whether the vignettes captured life with AD as seen by persons with the disease.

The vignettes may require adjustments for application to different populations. For example, the mild vignette describes a situation where someone interested in hockey might forget whether her or his favorite team won its last game. Researchers may wish to substitute baseball or (American) football for hockey in the United States, or (soccer) football for hockey in Europe. Similarly, some phrases may need revision to account for cultural understanding. A case in point is the phrase “too sick to be taken anywhere” in the severe vignette. ‘Too sick’ refers to being affected by AD to the point where an individual is bedridden, but ‘sick’ could refer specifically to vomiting in some cultures. Re-validation of the vignettes may be required, depending on the nature and extent of the adjustments.

The vignettes did not contain examples of stigma. Stigma can adversely affect outcomes such as depression, anxiety, physical health, self-esteem, social support, and social participation [43], as well as delay the diagnosis of AD [44]. Therefore, stigma can have a deleterious impact on the HRQoL of PwAD. Future research should assess the usefulness of incorporating stigma into the vignettes.

## Conclusions

The vignettes describe the mild, moderate, and severe health states of AD. The vignettes are intended to permit the general public to provide HRQoL estimates in AD. Few studies have explored the use of vignettes to measure HRQoL in any disease area [45]. Our research program studies a novel use of vignettes in the domain of HRQoL.

## Supporting Information

S1 File Interview Guide. (DOCX)
