# Supplementary material for: Development of Clinical Vignettes to Describe Alzheimer's Disease Health States: A Qualitative Study
Source: PLoS One. 2016 Sep 2;11(9):e0162422. doi: 10.1371/journal.pone.0162422 (PMC5010212; doi:10.1371/journal.pone.0162422)
Supplement: S1 File — (DOCX) [file pone.0162422.s001.docx]

**Interview Guide**

**Clinical Vignette 1 - Mild Alzheimer’s disease**

Please read the clinical vignette describing mild Alzheimer’s disease.

Does this vignette accurately describe mild Alzheimer’s disease based on your experiences taking care of your loved one or based on what you learned about this stage of disease from health professionals or any other information sources?

- 1. Do you feel persons with mild Alzheimer’s disease exhibit most of the symptoms described in this vignette?
  2. Are there any symptoms we should remove from this vignette?
  3. Are there any symptoms missing from this vignette that we should include?
  4. Alzheimer’s disease is more than symptoms alone. Some people adapt to the disease despite the cognitive, functional, behavioural, and psychological challenges they face on a daily basis. Do you have any suggestions about how we could include this idea of ‘adaptation’ in the vignette?

**Clinical Vignette 2 – Moderate Alzheimer’s disease**

Please read the clinical vignette describing moderate Alzheimer’s disease.

Does this vignette accurately describe moderate Alzheimer’s disease based on your experiences taking care of your loved one or based on what you learned about this stage of disease from health professionals or any other information sources?

1. Do you feel persons with moderate Alzheimer’s disease exhibit most of the symptoms described in this vignette?
2. Are there any symptoms we should remove from this vignette?
3. Are there any symptoms missing from this vignette that we should include?
4. Alzheimer’s disease is more than symptoms alone. Some people adapt to the disease despite the cognitive, functional, behavioural, and psychological challenges they face on a daily basis. Do you have any suggestions about how we could include this idea of ‘adaptation’ in the vignette?

**Clinical Vignette 3 – Severe Alzheimer’s disease**

Please read the clinical vignette describing severe Alzheimer’s disease.

Does this vignette accurately describe severe Alzheimer’s disease based on what you learned about this stage of disease from health professionals or any other information sources?

1. Are there any symptoms we should remove from this vignette?
2. Are there any symptoms missing from this vignette that we should include?
3. Alzheimer’s disease is more than symptoms alone. Some people adapt to the disease despite the cognitive, functional, behavioural, and psychological challenges they face on a daily basis. Do you have any suggestions about how we could include this idea of ‘adaptation’ in the vignette?
